# Dietary and Non-Dietary Modulators of Cognitive Function

**DOI:** 10.3390/nu15133015

**Published:** 2023-07-01

**Authors:** M. Hasan Mohajeri

**Affiliations:** Institute of Anatomy, University of Zurich, Winterthurerstrasse 190, 8057 Zürich, Switzerland; mhasan.mohajeri@uzh.ch; Tel.: +41-79-9381203

Manifold internal and external factors may influence brain function in the long run, including genetic predispositions as well as epigenetic and environmental factors. A healthy brain is of fundamental importance for the survival of animals. Therefore, it is intuitive that in highly evolved animals, such as mammals, very sophisticated mechanisms are in place to ensure normal, healthy conditions in the brain. This Special Issue focuses on factors that modulate brain function in health and disease. The objective of this editorial is to highlight and comment on important topics discussed in individual reports included in this Special Issue to showcase examples of dietary and non-dietary interventions impacting cognitive function.

The association of a high-fat diet with neuronal and memory dysfunction was the topic of a report by Huang and co-workers [1]. High-fat diets induce cognitive deficits in rodents that model human cognitive impairment [2]. The potential of dietary ingredients to influence the microbiome, thereby exerting varying levels of beneficial effects, is an active field of research [3]. The results presented here show that mice fed a high-fat diet supplemented with bilberry, blackcurrant, blueberry, lingonberry, and (to some extent) cloudberry exhibited better performance in cognitive tests [1]. Better cognition was linked to a greater proportion of doublecortin-expressing cells in the hippocampus. Furthermore, the proportion of the mucosa-associated symbiotic bacteria Akkermansia muciniphila was increased in the cecal microbiota of the supplemented mice. In this study, in contrast to previously reported data, hippocampal levels of BDNF were not altered [4,5]. BDNF expression may be dependent on age, diet, physical activity, and, notably, the brain region subjected to investigation, which may explain the differences between the studies. Altogether, these berries may be able to positively affect cognitive health.

In this regard, our team have systemically reviewed the mechanisms of action of microbiome-derived factors in the pathogenesis of common brain diseases [6]. We reported the contribution of the microbiota–gut–brain axis to the pathophysiology of seven brain-related diseases, namely attention deficit hyperactivity disorder, autism spectrum disorder, schizophrenia, Alzheimer’s disease, Parkinson’s disease, major depressive disorder, and bipolar disorder. Our central findings were that, mechanistically, all seven diseases are associated with a leaky gut, neuroinflammation, and over-activated microglial cells even if the involved bacteria differed depending on the studied disease. We have provided comprehensive detailed lists of pro-inflammatory shifts in patients’ microbiota, as well as alterations in the bacteria-derived metabolites associated with the different brain diseases including aberrant levels of short-chain fatty acids, neurotransmitters, and amino acids. When available, we have reported twin studies (the participants of which have the same genomic makeup) to be able to determine bacteria-related effects [7]. Consequently, alterations in the abundance of bacteria strains and altered bacterial metabolite levels have been defined that could likely be possible markers for disease diagnostics and could help to identify novel treatment options for brain-related disorders, underlining the necessity for a deeper understanding of the microbiota gut–brain axis.

The effects of high-phytate diets on Aβ accumulation and increased neuronal cell death were studied by one group of researchers in Sprague Dawley rats [8]. Rats fed high-phytate, low-calcium diets showed elevated accumulation of Aβ peptide and neuronal apoptosis in the cornu ammonis 3 (CA3) and dentate gyrus (DG) regions of the hippocampus. The amounts of insoluble Aβ in the treated hippocampi were increased, probably due to increased expression of *bace1* and *psen1*, which are critical genes for forming Aβ peptides. Thus, these data support the theory that being fed high-phytate diets might enhance amyloidogenic processing in rats due to transcriptional upregulation of the genes involved in the amyloidogenic processing of amyloid precursor protein (APP).

Whether changes in diastolic blood pressure over several years lead to changes in brain structure and cognitive function was studied by Takeuchi and Kawashima [9] in a large cohort from the UK Biobank (see https://www.ukbiobank.ac.uk/media/gnkeyh2q/study-rationale.pdf), including up to 502,505 participants. This report utilized follow-up data of approximately 10 years from the cohort in which the imaging dataset was collected at baseline and later during the time course of the follow-up period. The results of this association study showed that high baseline diastolic blood pressure could be linked to a slight improvement in reaction time and a slightly greater reduction in depression scores as well as greater total gray matter volume (GMV) retention. Aging itself showed an association with GMV reduction. A high cheese intake level at baseline could be associated with a reduction in diastolic blood pressure, a relative increase in depression scores, and a relative increase in fluid intelligence and visuospatial memory performance. The authors conclude that these results are in line with the view that higher BP in the aging brain has a complex role.

A prospective study by the same researchers utilizing data from the above cohort aimed to answer the question of whether nutrition and dietary patterns in middle- to old-aged European adults are linked to the development of dementia [10]. The association between the consumption of basic food categories and the risk of incident dementia was analyzed in around 500,000 participants at baseline and after a follow-up period of up to 10 years. The collected data demonstrated that moderate total meat and total fish intake, low vegetable and fruit intake, and high bread intake were found in subjects who are less likely to develop dementia later in life. These results contrast greatly with some previously published reports and the notion that the Mediterranean diet reduces the risk of dementia [11]. Future investigational studies are needed to confirm causality.

Finally, Satyam and Bairy [12] reviewed the bioavailability, off-target effects, and mechanism of action of nutraceuticals to fight neuroinflammation. The authors mention that naturally derived substances to improve brain health are generally called “neuro-nutraceuticals” [13]. Limited clinical data, however, are available to date to substantiate the safety, efficacy, and possible drug–food and drug–drug interactions of neuro-nutraceuticals. Inflammation is a crucial factor in aging and chronic degenerative diseases. Therefore, the above authors explored the key molecular cues influencing the neuroinflammatory processes and neuro-nutraceuticals positioned to combat neuroinflammation and highlighted translational challenges associated with neuro-nutraceuticals. The results showed that the therapeutic application of neuro-nutraceuticals is restricted because of limited knowledge on the complex metabolic pathways/activities, the possibility of drug–drug interaction, and the possible involvement of the gut microbiota in the bioconversion of phytochemicals [12]. The authors concluded that the side effects of neuro-nutraceuticals need to be fully understood in order to prevent negative serious side effects and emphasized the necessity of conducting additional research before recommending neuro-nutraceuticals for the treatment of neuroinflammatory brain diseases.

In summary, the optimal functioning of the brain may be compromised by various internal or external factors during daily life. Any disruption of brain physiology that overwhelms the repair and compensatory mechanisms of the brain may potentially lead to a diseased status. Because of their methodology, epidemiological studies can only provide supportive correlations between the cause and consequence of a given disease, whereas clinical studies are normally designed to examine the effect of a factor on one (or a few) predefined endpoints. As such, a combination of both study designs is needed to determine the mechanisms of compounds affecting the brain. Ideally, further intensification of both types of research will result in drafting meaningful guidelines for maintaining healthy brain function and for the diagnosis and treatment of brain-related conditions.
